# Bibliometric Analysis of the Research Status and Global Trends in Behavioral and Psychological Symptoms of Dementia in Alzheimer’s Disease from 2002 to 2022

**DOI:** 10.2174/1570159X21666230807144750

**Published:** 2023-08-24

**Authors:** Haipeng Cai, Ruonan Du, Kebing Yang, Wei Li, Zhiren Wang

**Affiliations:** 1 Beijing Huilongguan Hospital, Huilongguan Clinical Medical School of Peking University, Beijing, China

**Keywords:** Alzheimer’s disease, behavioral and psychological symptoms of dementia, bibliometrics analysis, CiteSpace, global trends, cognitive impairment

## Abstract

**Background:**

Several reviews on behavioral and psychological symptoms (BPSDs) in patients with Alzheimer’s disease (AD) have summarized the current state of this field, but global trends are unclear.

**Objective:**

This study utilized CiteSpace to provide a global overview of the current state of research on AD and its BPSDs and to predict future research trends in the field.

**Methods:**

Data were retrieved from the Web of Science Core Collection. Bibliometric and co-occurrence analyses were performed using CiteSpace software. In total, 787 valid publications were included in the analysis.

**Results:**

Publications on AD and BPSD have shown an increasing trend since 2002. The United States and the University of Toronto were the countries and institutions with the highest total number of publications, respectively. Japan and China were the second and third most influential in the field. Clive Ballard was the top author in terms of the number of publications. Journal of Alzheimer's Disease had the highest number of publications on this topic. Co-occurrence analysis showed that AD, behavioral symptoms, cognitive impairment, and early markers are hot topics in this area. Non-drug management of BPSDs, pharmacological treatment, and physiotherapy will be a hot topic in this field in the future.

**Conclusion:**

Our study visualized the relevant articles over the past 21 years to detect global hotspots and trends. Our findings may help researchers to identify research hotspots in this field and will help in the selection of appropriate research topics, while possibly leading to cross-regional cooperation.

## 1 INTRODUCTION

Alzheimer's disease (AD) is a progressive neurodegenerative disorder characterized by the deposition of amyloid-β plaques and hyperphosphorylated tau tangles [1]. Twelve risk factors, including low education, high blood pressure, excessive alcohol consumption, smoking, obesity, depression, and air pollution, increase the risk of dementia [2]. The increasing aging of the population and the growing number of people with dementia worldwide constitute a serious public health problem. According to the 2018 International Alzheimer's Disease Report, approximately 50 million people worldwide have dementia and this number is predicted to exceed 152 million by 2050 [3].

AD is the main cause of dementia [4, 5] and accounts for approximately 50-75% of all cases of dementia [6]. The early presentations of AD are mainly near-memory loss and personality changes. As the disease progresses, cognitive functions, such as a decline in language function, visuospatial impairment, inattention, and gradual loss of social functions eventually lead to an inability to care for oneself and the need for dedicated care. Along with an overall decline in cognitive function, more than 90% of patients develop behavioral and psychological symptoms (BPSDs), with apathy, with an overall prevalence of 49%, being the most common symptom [7]. This is followed by depression, aggression, anxiety, and sleep disturbances. Other symptoms, such as irritability, appetite disturbances, abnormal motor behavior, delusions, hallucinations, and euphoria, are less common, with prevalence rates ranging from 16% to 36% in AD cases [7]. BPSDs not only cause severe emotional distress in patients and their caregivers but are also associated with reduced quality of life. It also increases the economic burden on the patient's family and on society [8].

Non-pharmacological treatment is usually recommended as the first-line treatment option for BPSDs [9]. This mainly includes moderate exercise [10], training for different cognitive domains (memory, language function, visuospatial ability, executive function, and attention), lifestyle interventions, music therapy, and aromatherapy [11, 12]. However, the use of non-pharmacological interventions alone in the clinical setting does not completely alleviate BPSDs, and pharmacological interventions (such as antipsychotics, antidepressants, mood stabilizers, and cholinesterase inhibitors) are often required; therefore, balancing efficacy and tolerability is an important issue in clinical medication use.

Bibliometric analysis, first proposed by Pritchard, has clear advantages in reflecting the current situation and predicting future trends [13]. Bibliometrics can fill this gap by analyzing publications to assess the contributions of countries, institutions, and authors in the field qualitatively and quantitatively and predict future research trends [14]. In recent years, bibliometrics has been widely used in the medical field [15]. This study used literature data from the Web of Science Core Collection (WOSCC) for bibliometric and visualization analysis. BPSD was statistically calculated with respect to AD-related literature and the results were visualized.

Currently, several reviews on BPSDs in patients with AD have summarized the current state of the field [16-18], but global trends in the field are unclear. This study can fill the gap in this area and explore the development of this field. This study aims to help researchers find suitable journal publications and research partners by analyzing countries, regions, authors, keywords and citations and also helps to identify current research hotspots and future trends of BPSD in AD patients and provide further perspectives on the treatment of BPSD.

## 2 MATERIALS AND METHODS

### 2.1 Data Sources

**Fig. (1):**
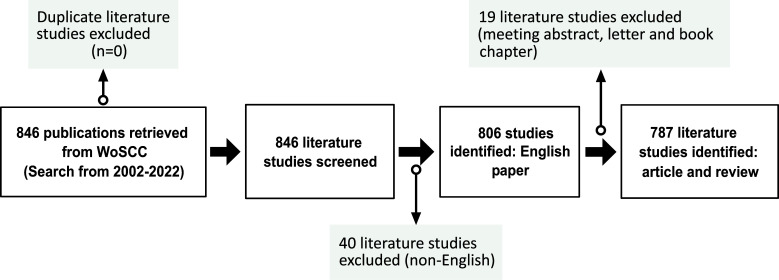
Flow chart of the study.

The data for this study were retrieved from the Web of Science Core Collection (WoSCC), which includes several datasets and is one of the most commonly used databases for bibliometric analyses. We conducted a search of the WoSCC database on December 31, 2022 (Fig. **1**), using the parameters below:

Search strategy: (TS=Behavioral and Psychological Symptoms of Dementia) and (TS=Alzheimer’s disease).

Search time range: 2002-2022.

Literature type: Articles and Reviews.

Language: English

The results of the search were articles in which the above subject terms appeared in the title, abstract, and keywords. We excluded “meeting abstract”, “book chapter”, and “correspondence” publications. The remaining 787 papers were mostly composed of original articles and reviews.

### 2.2 Introduction of Analysis Tools and Data Analysis

CiteSpace is a Java program developed by Chaomei Chen to visualize literature data from databases. The software is used to analyze trends in the evolution of disciplinary research frontiers, as well as internal links between different research frontiers. It also helps to identify core countries/ regions, institutions, and authors and their collaborations. Co-occurring keywords can reveal research foundations and hotspots. Detecting bursts can help researchers to predict research trends. Centrality is a key indicator for analyzing the importance of keywords, and if the centrality of a node exceeds 0.1, it indicates that the node is more important and has more influence on the research. The connection lines between nodes in the visualization represent the collaboration between institutions. The larger the node, the more content has been published. A change in color indicates different publication dates, with colors closer to purple indicating an earlier publication date and those closer to yellow indicating that the article was published recently.

We saved the literature data downloaded from WoSCC in a plain text format and used CiteSpace v6.1 R6 (https://citespace.podia.com/download) for data analysis. The specific parameters were set as follows: time slice: January 2002-December 2022, years per slice=1. Term source: Title, Abstract, Author keywords and Keywords, Node type: author, institution, country, keyword, citation, citation author and citation journal; link strength: Cosine, Selection criteria: Top N = 50; g-index = 25; Pruning: MST.

After completing the above settings, click the “Go” button for visualization analysis. The output of visualization is related to the selected node type. The visualization results (the presentation of nodes, lines, labels, *etc*.) could be adjusted in the panel. It could get burst references and keywords by setting the parameters of “Burst” in the panel. Under the “Node” menu, there is a button to calculate the centrality of nodes.

## 3 RESULTS

### 3.1 Distribution of Annual Publication Numbers

**Fig. (2):**
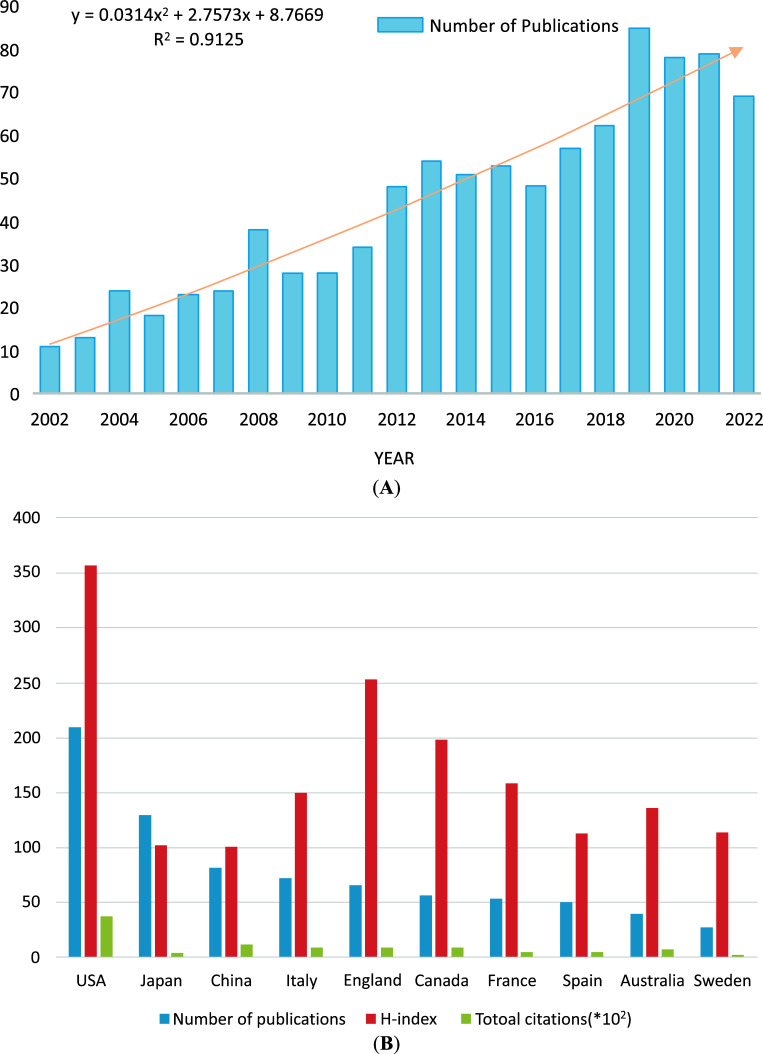
(**A**) The global distribution of countries of the research. (**B**) The number of publications from 2002 to 2022.

A change in the number of research papers is an important indicator of trends in a field. Since 2002, the number of publications on BPSDs and AD has shown an increasing annual trend, with 787 relevant articles published as of December 31, 2022 (articles and reviews), indicating a high level of research on BPSDs in AD patients. Excel software (Microsoft Corp., Redmond, WA, USA) was used to predict the number of publications. The predicted growth model equation was y = 0.0162x^2^ + 2.5421x + 6.9669, R^2^ = 0.8505, with x representing (forecast year - 2002) and y representing the predicted number of publications per year (Fig. **2A**). It is expected that the number of annual publications in this field could reach ∼95 or more by 2030.

### 3.2 Contribution and Quality of the Publications of Each Country

Sixty-nine countries have published articles on BPSDs and AD. The United States had the highest number of publications in this area (n = 209; 26.56%), followed by Japan (n = 129; 16.39%), and China (n = 80; 10.17%). The three countries with the highest number of publications include two East Asian countries, which may be related to the increasing aging of the population in both countries.

The h-index is an indicator commonly used to assess the academic impact of scholars, and it is defined as h of the N papers published by scholars, each of which has been cited at least h times. This index is now also used to assess the academic impact of research groups, such as research institutions and countries/regions. The US had the highest h-index (357), followed by the UK (253), and Canada (198). In addition, the US had the highest number of total citations (3794), followed by China (1126) (Table **1** and Fig. **2B**).

### 3.3 Analysis of Scientific Collaboration Network

**Fig. (3):**
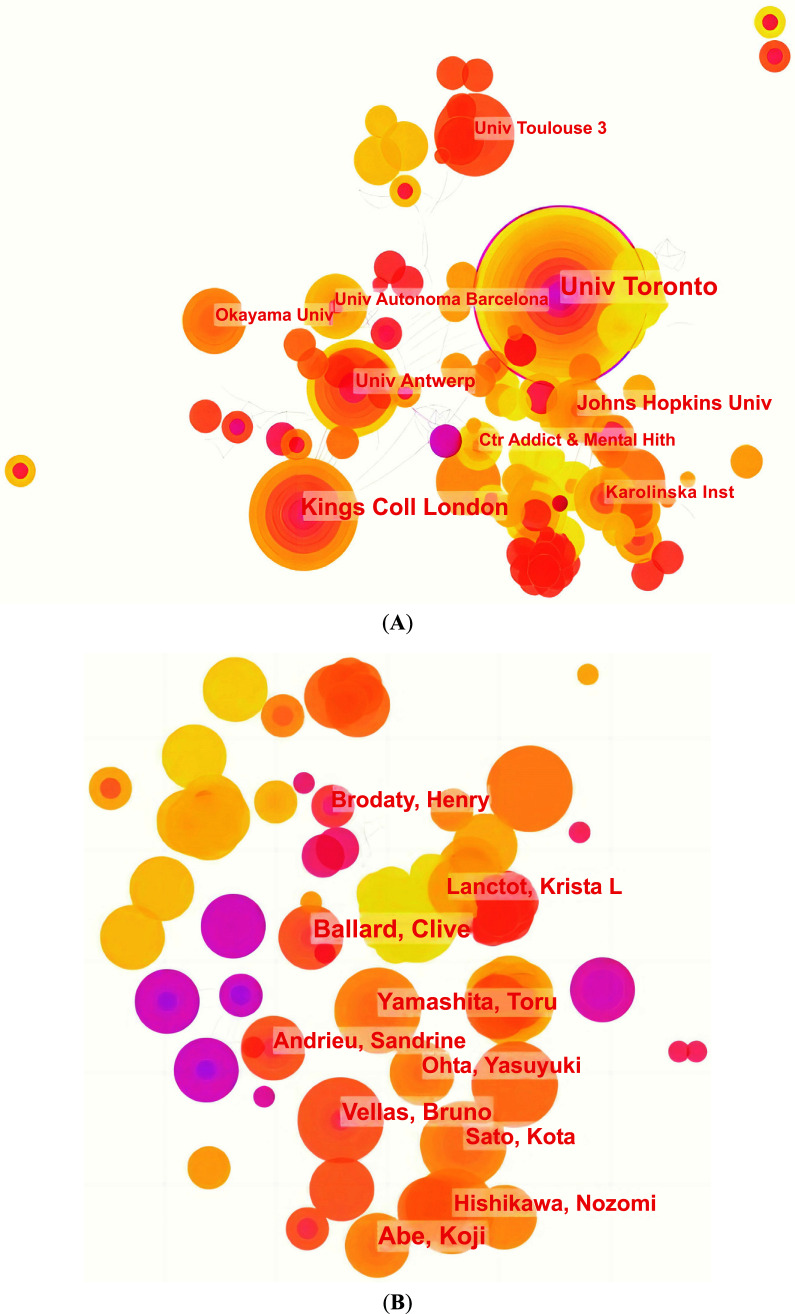
(**A**) Collaboration among institutions. (**B**) Collaboration among authors.

In the past 21 years, 547 institutions have published in this field. Table **2** lists the top 10 institutions by publication and centrality. The top-3 institutions contributing the most to the field were the University of Toronto, King's College London, and Johns Hopkins University. The top-3 institutions in terms of centrality were the Center Addict & Mental Health, University of Antwerp, and University College London. Based on an analysis of publications and centrality, the University of Toronto has 35 publications. The density of the institutional collaboration network was 0.0014, with 548 nodes and 204 links (Fig. **3A**), indicating that collaboration between institutions needs to be strengthened.

Table **2** lists the top 10 authors based on publications and centrality, with three authors having published more than 10 articles in the field as core authors. The author with the highest number of publications during the analyzed period was Clive Ballard. Fig. (**3B**) shows the collaboration network among authors, which contained 652 nodes and 1,107 connections, with a density of 0.0052, with some collaboration among top researchers in the field. However, the intensity of collaboration is low, and there is a need for greater cooperation among researchers from different countries, such as cross-national collaboration or multi-center research.

### 3.4 Analysis of Journals and Co-cited Journals and Co-cited References

Articles on BPSDs and AD were published in 292 journals during the analyzed period. The top 10 journals and co-cited journals mainly involved the fields of geriatrics, psychiatry, and behavioral medicine (Table **3**). The journal with the highest number of publications in this period was the Journal of Alzheimer's Disease, followed by International Psychogeriatrics and International Journal of Geriatric Psychiatry, and five of the top 10 journals in terms of publication number had impact factors above 5.00. The analysis of co-cited journals shows the contribution of each journal to the field. The top-ranked journal according to the co-citation count is *Neurology* (590), and it is the official publication of the American Academy of Neurology (AAN), a highly prestigious and established journal in the field of neurology with high quality. The International Journal of Geriatric Psychiatry (562) and International Psychogeriatrics (497) were ranked second and third, both are in the field of geriatrics.

Table **4** shows the details of the top 10 highly cited articles. Three of these articles had HC Kales as the first author and focused on how to manage BPSDs, with the most recent publication being a Delphi Consensus on non-pharmacological and pharmacological treatments [19]. The Delphi Consensus is a structured communication technique or method that relies on a systematic, interactive predictive approach by a panel of experts to narrow the range of answers discussed and gradually move toward a consensus “correct” answer. The consensus results showed a clear tendency to escalate the management of BPSDs in patients from identifying the underlying cause.

The analysis of co-cited journals and co-cited articles showed that most of the articles were published in journals in the field of geriatrics and psychiatry with high impact factors, including many top journals such as *NEMJ* and *JAMA*. This also suggests to us that the issue of targeting psycho-behavioral symptoms in patients with dementia is one of the current hot topics of concern in geriatrics worldwide. Therefore, we speculate that high-quality studies in this area are expected to be published in top journals, which also requires us to further check the quality of future studies and strengthen the basic and theoretical research.

### 3.5 Analysis of Burst Keywords and Citation

**Fig. (4):**
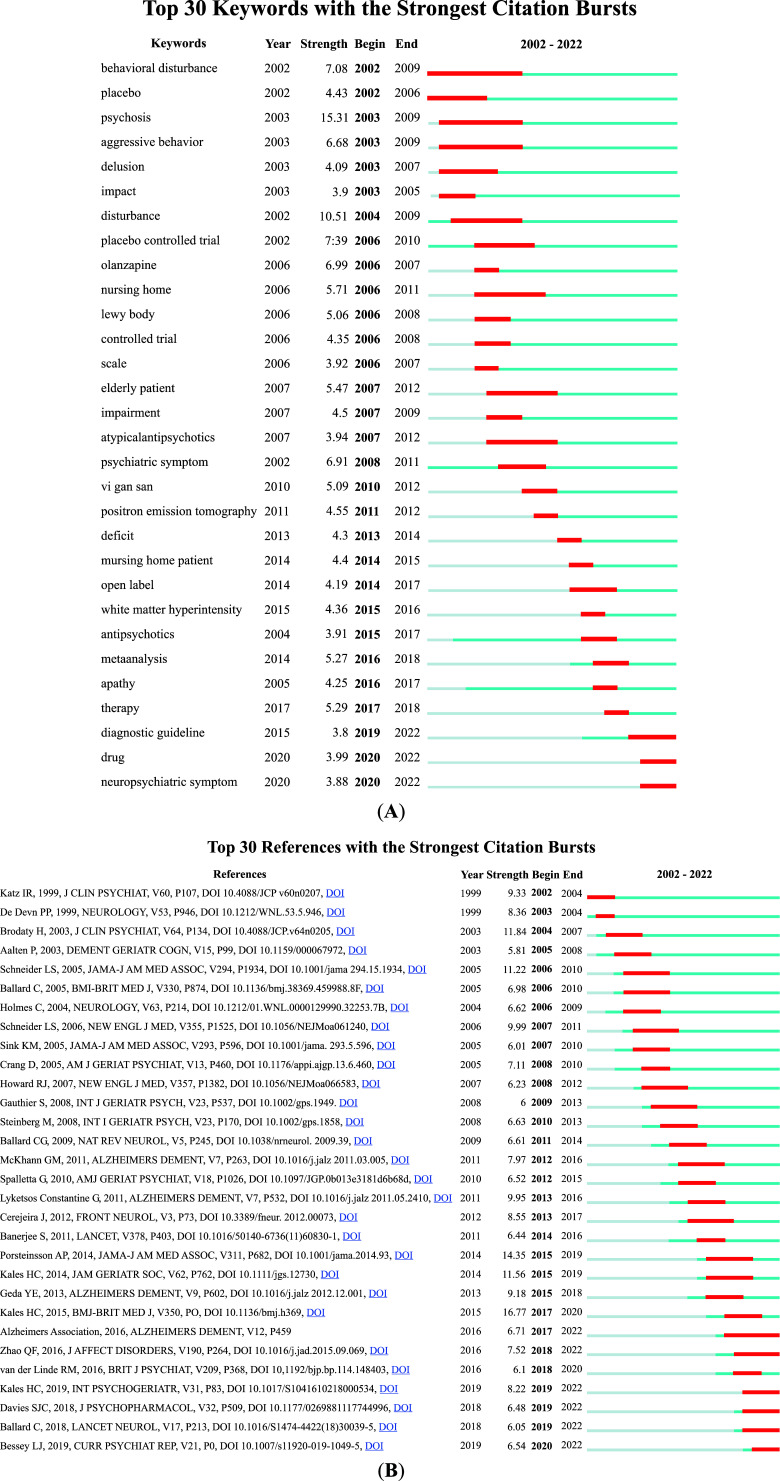
(**A**) Top 30 keywords with the strongest citation bursts (**B**) Top 30 references with the strongest citation bursts.

Keyword bursts reflect hot topics and trends in a research field during a certain period of time. The blue line represents the last 21 years and the red line represents the duration of the keyword burst, demonstrating the evolution of hot topics. Fig. **4A** shows the top 30 most cited keywords. “Behavioral disturbance” was the keyword with the longest burst duration in this field from 2002 to 2009, with a strength of 7.08. “Psychosis” was the strongest keyword for the last 21 years (strength: 15.31). The keywords that lasted until 2021 mainly included diagnostic guidelines, drugs, and neuropsychiatric symptoms, which reflect the latest research trends.

The citation bursts of references illustrate the evolution of the knowledge domain. Fig. **4B** lists 30 references with high citation volumes. The duration of citation bursts is shown in the red line segments. The latest citation bursts of references involved papers published in 2019 with intensities of 8.22 [19] and 6.54 [20], respectively. Six references had bursts that lasted until 2022 [7, 19-23]. The strongest citation burst, with an intensity of 16.77 [24], focused on the assessment and management of BPSDs in community-dwelling patients with dementia, and was published in 2015.

### 3.6 Analysis of Co-occurring Keywords

Table **5** lists the top 10 keywords according to their frequency of occurrence. The top 2 most frequently occurring keywords were “Alzheimer's disease” and “psychological symptom”. The next most frequent keyword was “dementia.” Subsequently, we clustered the co-occurring keywords to find the hot topics of the study. In general, we considered the clustering results reliable when the silhouette was > 0.7. Using the LLR clustering method, we obtained four clusters with silhouette > 0.8 and seven clusters with silhouette > 0.7 (Table **6**). Among them, cluster#0, which has the largest size value, was considered reliable in terms of clustering results, although the silhouette value was 0.597. According to the co-occurring keywords and clustering results, Alzheimer's disease, behavioral symptoms, cognitive impairment, and early markers were popular research topics. We found that the results of the clustering were broadly divided into three areas: disease management related to quality of life, pharmacology related to drug therapy, and diagnostic-related indicators. This is roughly the same as the results for burst keywords and citations, and therefore we speculate that this is a hot spot for future research.

## 4 DISCUSSION

No previous bibliometric analysis has been applied to the study of BPSDs in AD patients. Our study visualized the relevant articles over the past 21 years to detect global hotspots and trends.

### 4.1 Global Trends in BPSDs of AD

Our analysis shows that the number of publications in this field has increased since 2002. Among many countries/ regions, the US had the highest number of publications on this topic, contributing significantly to the field. The University of Toronto had the highest number of publications in the field for the last 21 years, followed by King's College London and Johns Hopkins University. Clive Ballard was the author with the highest number of publications on this topic and collaborated with other researchers. Japan and China, the second and third most-published countries in this field, respectively, have contributed to and influenced more research in this field in recent years, which we speculate is related to the gradual aging of the population in both countries. We also found a low density of collaborative networks between different countries and institutions, suggesting that collaboration between countries or institutions needs to be strengthened. Our study may help researchers find different partners.

### 4.2 Status of Publications

This study found that most of the articles in this field were published in journals related to psychiatry, geriatrics, and behavioral medicine, and the impact factors of the journals were high, indicating that the research in this field is of high quality and that the findings are reliable. The Journal of Alzheimer's Disease is the journal with the most publications on this topic, and it receives articles related to the etiology, pathogenesis, epidemiology, genetics, and behavior of AD, allowing readers to understand the progress in AD research from multiple disciplines. Four of the 10 most-cited papers overall were published in top journals, such as *JAMA.* Most of these papers deal with the management of BPSDs and research on the pharmacological treatment of BPSDs.

The most co-cited literature is the article by Porsteinsson *et al.* [25], published in *JAMA.* The primary objective of their study was to evaluate the efficacy of citalopram in the treatment of agitation in patients with AD. They randomized 186 patients with AD into a citalopram and placebo group for a 3-week drug treatment (the dose of citalopram was titrated up from 10 mg/day to a maximum dose of 30 mg/day, depending on the patient). The psychosocial intervention was administered to both groups, and patients' agitation was measured using the Neurobehavioral Rating Scale agitation subscale, the modified Alzheimer's Disease Cooperative Study-Clinical Global Impression of Change, and other scales to assess patients' treatment outcomes. The results showed that citalopram relieved agitation and reduced caregiver burden in patients with AD, as compared to placebo, but the drug side effects (cognitive or cardiac side effects) of citalopram placed an upper limit on its dosage.

In addition to studies of antidepressants for BPSDs, the efficacy, and safety of antipsychotic medications for BPSDs have also been studied. The results of a randomized, double-blind, controlled placebo trial by Brodaty *et al.* [26] showed that low doses of risperidone (mean 0.95 mg/day) significantly improved aggression and agitation in patients with dementia. They also found that the probability of drowsiness and urinary tract infections was higher in patients with dementia in the risperidone group than in those in the control group. This suggests that, although atypical antipsychotics can improve BPSD symptoms in patients with dementia, their safety is also a key consideration in clinical practice.

Schneider *et al.* [27] expanded the drug selection by comparing the efficacy of olanzapine (mean dose 5.5 mg/d), quetiapine (mean dose 56.5 mg/d), and risperidone (mean dose 1.0 mg/d) for the treatment of BPSDs in patients with AD with placebo and assessed their safety. They found no significant difference in the time to discontinuation of treatment for any reason among the four groups, and the Clinical Global Impressions assessment of the four groups showed no significant difference in efficacy between the four groups. However, the time to treatment discontinuation due to adverse events or intolerance to treatment was biased toward a placebo. This suggests that both placebo and antipsychotics provide some improvement in BPSDs in patients with AD, but a placebo is less likely to cause adverse events and is safer. A meta-analysis [28] also indicated that atypical antipsychotics may be associated with a small increase in the risk of death as compared with a placebo, which also suggested that there are risks for clinicians using antipsychotics for BPSDs and that patients' somatic conditions need to be fully considered before using these drugs.

### 4.3 Current Research Hotspots and Frontiers in the Future

By reading and sorting out the bursts in terms of keywords and citation results, we gain an outlook on the possible future research hotspots in this field.

#### 4.3.1 Non-drug Management of BPSDs in AD Patients

BPSDs in dementia patients mainly include symptoms of aggression, agitation, depression, hallucinations, and apathy, which not only affect the entire course of dementia, leading to a high morbidity and mortality rate in these patients [29], and pose a threat to the caregivers' own health and quality of life [30], while also increasing the socioeconomic burden [31]. Therefore, the management of BPSDs in patients with dementia is one of the main research focuses.

Because antipsychotics significantly increase the risk of death in patients with AD, the US Food and Drug Administration officially issued a black box warning [32] prohibiting the use of atypical and typical antipsychotics for the treatment of dementia-related psychiatric or behavioral disorders. Non-pharmacological treatment is currently used clinically as the first-line treatment for BPSDs, which mainly includes cognitive/emotional memory interventions (situational therapy, reminiscence therapy, *etc*.), sensory stimulation interventions (music therapy, massage therapy, *etc*.), and other psychosocial interventions [17]. The efficacy of non-pharmacological treatments is recognized as effective, and a meta-analysis showed that non-pharmacological interventions not only reduced the frequency and severity of BPSDs in AD patients but also reduced caregiver distress [33]. However, not all patients with AD have access to professional non-pharmacological interventions, and a proportion of patients rely on family caregivers (relatives or non-professionals) for their care, which is likely to exacerbate their BPSDs if inappropriate care is used, and can put stress on their own health. Thus, they need some supportive interventions. A recent meta-analysis showed that Internet-based family-caregiver interventions have potential benefits for BPSDs and can improve caregiver health outcomes [34]. This also suggests that we should not neglect the health status of caregivers when caring for people with AD.

Effective non-pharmacological treatments have the potential to alter the course of AD, reduce healthcare costs, and lower the economic burden on families and society while improving the quality of life for patients and caregivers. However, further exploration is needed on how to manage BPSDs in patients with AD better, taking into account the physical and mental health of caregivers. Therefore, we predict that the non-drug management of BPSDs in AD will be a hot topic for future research.

#### 4.3.2 Pharmacological Treatment

In actual clinical practice, individually customized nonpharmacological interventions are effective for AD patients with milder BPSDs [35, 36]. However, non-pharmacological interventions alone are not enough, pharmacological treatment is equally important. For BPSD symptoms, doctors first choose medications that improve cognition, such as donepezil, memantine, and carboplatin bitartrate. The use of these drugs alone or in combination with two or more treatments can improve not only the BPSD symptoms but also the cognitive symptoms in patients with dementia. Unfortunately, the nootropic drugs do not improve all BPSD symptoms such as agitation, euphoria, hallucinations/delusions, *etc*.

For symptoms or behaviors that are difficult to treat with non-pharmacological interventions, clinicians usually choose psychotropic medications, and most times in reality, caregivers request medication. Ballard and Corbett [37] summarized several randomized controlled trials (RCTs) that evaluated the treatment outcomes of atypical antipsychotics. They found that antipsychotic medications were significantly effective primarily in the treatment of aggression symptoms, but had limited efficacy for other BPSDs and provided no additional benefit for patient outcomes with more than 12 weeks of continuous medication. In addition, the use of atypical antipsychotics in this population is associated with significant adverse effects, including sedation, extrapyramidal symptoms, gait disturbances, falls, cardiovascular events (postural hypotension, arrhythmias, prolonged QTc interval, *etc*.), cerebrovascular events, and even death [28, 37]. The results of a meta-analysis by Yury *et al.* [38] also showed a moderate effect size for atypical antipsychotics for BPSDs, and no statistically or clinically significant differences between atypical antipsychotics and placebo. However, other meta-analyses came to a different conclusion: Jin *et al.* [39] analyzed 146 RCTs and found that antipsychotics (aripiprazole, risperidone, haloperidol, and quetiapine) were significantly more effective than placebo for BPSDs. Although adverse effects, such as diarrhea, dizziness, and cerebrovascular events, occurred, the safety was within acceptable limits. Hence, they concluded that pharmacotherapy should be the treatment of choice for BPSDs.

In fact, some medication decisions for BPSDs are reasonable and necessary; therefore, some guidelines state that long-term use of antipsychotic medications should be avoided and can be discontinued when symptoms are controlled as appropriate [40]. However, further studies are needed to determine whether the quality of life and mortality of patients are affected by discontinuation of antipsychotics [41]. The above results suggest that we must fully assess the underlying somatic condition of patients before using antipsychotic drugs in AD patients and must obtain the consent of patients' guardians to use them vigilantly, with careful assessment of efficacy and safety pros and cons.

Due to the specificity of the patient population, no national pharmacovigilance authority has approved antipsychotic medication for BPSD, and there are a limited number of RCTs on the pharmacological treatment of BPSDs, with most studies focusing on the efficacy of drugs for individual BPSDs (such as apathy, anxiety, and agitation), and with inconclusive results. How to choose antipsychotic drugs? Which medication is more effective to choose for different BPSD symptoms? Do different medications need to be selected at different stages of dementia? How long does the use of antipsychotics for BPSD last? What are the benefits to patients and caregivers of stopping use? There is a paucity of research in these areas. These commonly encountered clinical questions require further research, and therefore we predict that antipsychotic treatment of BPSD symptoms in AD will be one of the directions of future research.

#### 4.3.3 Physiotherapy

The results of a growing number of studies have suggested that physical therapy, such as electroconvulsive therapy (ECT) and transcranial magnetic stimulation (TMS), are effective methods for alleviating BPSDs. Hermida *et al.* [42] conducted a retrospective analysis of 60 patients with dementia who were treated with ECT to explore the effectiveness of ECT on agitation symptoms. They found that agitation was reduced, the dosage of psychotropic medications was decreased, and the overall level of functioning was elevated. Although six patients experienced postictal agitation and transient impairment of consciousness after the first treatment, oral administration of a small dose of olanzapine 30 min before each subsequent treatment prevented these side effects, and no other side effects were observed for the time being. In a review of 20 published articles (retrospective studies and case reports) on ECT for BPSDs, Tampi, *et al.* [43] concluded that ECT appeared to be a safe treatment for older adults, with few contraindications and transient adverse effects. Therefore, ECT may be a treatment option in BPSDs management strategies. In clinical practice, guardians are concerned about whether ECT will reduce cognitive function in patients with dementia and may refuse treatment.

Although studies have shown that ECT is safe and there is no evidence at this time that ECT further impairs cognition in people with dementia [44, 45], guardians or caregivers still have concerns about this and may refuse the treatment or research on the treatment. In addition, studies on ECT for BPSD have been limited in terms of age and somatic condition, and for these reasons, the sample size of current studies is relatively small. Therefore, studies with large sample sizes are needed if we want to investigate the specific parameters of ECT that are effective for BPSD (*e.g*., power level, duration) or which BPSD symptoms ECT is more effective for. Based on this, we believe that ECT for BPSD will be one of the hot spots for future research. A study by William *et al.* suggested that nonconvulsive electrotherapy (NET) is effective for depressive symptoms [46], based on which we hypothesized that NET for BPSD may also be effective and that NET may be safer and more acceptable to guardians for patients with dementia, but this needs to be verified by future relevant studies.

In addition, Vacas *et al.* [47] performed a meta-analysis of the literature on repetitive transcranial magnetic stimulation (rTMS) and transcranial direct current stimulation, but the limited number of valid RCTs included in this study only suggests that we have some effectiveness of rTMS for BPSDs; therefore, more clinical studies need to be included in the analysis to draw more definitive conclusions.

As research has unfolded, evidence of the effectiveness of physical therapy for BPSDs has grown, and most studies have affirmed the safety of physical therapy. Therefore, we speculate that future studies should continue to focus on this direction as well.

## 5 LIMITATIONS

This study had some limitations. First, the importance of recent publications may have been underestimated due to their low citation counts. Second, we only searched the literature in English and ignored publications in other languages, possibly missing some contributions from non-English-speaking countries.

## CONCLUSION

Since 2002, there has been an increasing trend in the number of publications, with the United States and the University of Toronto being the most influential countries and institutions, respectively, while Japan and China also have a marked presence in the field. Clive Ballard made a large contribution to the field as the author with the highest number of publications. The Journal of Alzheimer's Disease had the highest number of publications. The above results help researchers to find research partners as well as matching journal publications. Combining the burst analysis of keywords and references, we predicted the possible directions of future research. First, BPSD not only causes distress in the lives of the patients themselves but also increases the burden of life for caregivers; therefore, we predict that the treatment of BPSD is a hot topic for future research. Current guidelines preferentially recommend non-pharmacological interventions, but there is no conclusive evidence as to what non-pharmacological interventions are more effective for the sub-symptoms of BPSD and what medications will work for BPSD symptoms in patients for whom non-pharmacological treatments have failed, and we predict an increase in research on non-pharmacological and pharmacological interventions. Whether some physical therapy approaches will work for BPSD in patients with dementia also needs to be explored in the future. In addition, we also suggested the possibility of NET for BPSD, which also needs our future empirical study. The results of this study may lay the foundation for future studies.

## Figures and Tables

**Table 1:** The top 10 productive countries of the topic.

**Country**	**Number of Publications**	**H-index**	Totoal Citations (*10^2^)
USA	209	357	37.94
Japan	129	102	4.00
China	80	101	11.26
Italy	72	148	9.16
England	66	253	9.00
Canada	56	198	8.69
France	54	159	4.73
Spain	50	112	4.65
Australia	40	136	7.02
Sweden	28	114	2.97

**Table 2:** Top 10 institutions, and authors according to publications and centrality.

**Items**	**Publications**			**Centrality**		
	**Ranking**	**Name**	**Number**	**Ranking**	**Name**	**Number**
Institution	1	University of Toronto	35	1	Center Addict & Mental Health	0.14
	2	King’s College London	23	2	University of Antwerp	0.13
	3	Johns Hopkins University	15	3	University College London	0.11
	4	University of Antwerp	14	4	King's College London	0.10
	5	Karolinska Institute	12	5	Keio University	0.10
	6	Chang Gung University	11	6	University of Toronto	0.09
	7	University of Autonoma Barcelona	11	7	Louis Pasteur University	0.09
	8	Center Addict & Mental Health	10	8	Kyoto University	0.08
	9	University of Toulouse	10	9	Kumamoto University	0.08
	10	Okayama University	10	10	Johns Hopkins University	0.08
Author	1	Clive Ballard	12	1	Clive Ballard	0.04
	2	Abe Koji	12	2	Lanctot L Krista	0.03
	3	Vellas Bruno	11	3	Brodaty Henry	0.03
	4	Sato Kota	9	4	Ismail Zahinoor	0.03
	5	Hishikawa Nozomi	9	5	Black E Sandra	0.02
	6	Yamashita Toru	9	6	De deyn Peter Paul	0.02
	7	Sandrine Andrieu	8	7	Kumar Sanjeev	0.02
	8	Lanctot L Krista	8	8	Engelborghs Sebastiaan	0.01
	9	Brodaty Henry	8	9	Ames David	0.01
	10	Yasuyuki Ohta	8	10	Lyketsos G Constantine	0.01

**Table 3:** Top 10 journals and co-cited journals.

**Items**	**Ranking**	**Name**	**Counts**	**IF(2022)**
Journal	1	*Journal of Alzheimer’s Disease*	93	4.160
	2	*International Psychogeriatrics*	62	7.191
	3	*International Journal of Geriatric Psychiatry*	38	3.850
	4	*Dementia and Geriatric Cognitive Disorders*	23	3.346
	5	*Psychogeriatrics*	21	2.295
	6	*American Journal of Geriatric Psychiatry*	20	7.996
	7	*Frontiers in Pharmacology*	18	5.988
	8	*Aging and Mental Health*	17	3.514
	9	*American Journal of Alzheimers Disease and Other Dementias*	16	5.361
	10	*Journal of the American Medical Directors Association*	16	7.802
Co-cited Journals	1	*Neurology*	590	11.800
	2	*International Journal of Geriatric Psychiatry*	562	3.850
	3	*International Psychogeriatrics*	497	7.191
	4	*American Journal of Geriatric Psychiatry*	452	7.996
	5	*Journal of the American Geriatric Society*	440	7.538
	6	*Dementia and Geriatric Cognitive Disorders*	398	3.346
	7	*American Journal of Psychiatry*	393	12.759
	8	*JAMA*	353	157.335
	9	*Journal of Alzheimer’s Disease*	314	4.160
	10	*Alzheimers Dement*	308	16.655

**Table 4:** Top 10 highly cited references.

**Ranking**	**Cited by**	**Author**	**Title (Publication Year)**	**Journal (IF2022)**
1	38	Porsteinsson, AP *et al.*	Effect of Citalopram on Agitation in Alzheimer Disease. (2014)	*JAMA* (157.335)
2	36	Kales HC *et al.*	Assessment and management of behavioral and psychological symptoms of dementia. (2015)	*BMJ* (93.333)
3	28	Kales HC *et al.*	Management of neuropsychiatric symptoms of dementia in clinical settings: recommendations from a multidisciplinary expert panel. (2014)	*Journal of the American Geriatric Society* (7.538)
4	24	Brodaty H *et al.*	A randomized placebo-controlled trial of risperidone for the treatment of aggression, agitation, and psychosis of dementia. (2003)	*Journal of Clinical Psychiatry* (5.906)
5	24	Schneider LS *et al.*	Risk of death with atypical antipsychotic drug treatment for dementia: a meta-analysis of randomized placebo-controlled trials. (2005)	*JAMA* (157.335)
6	22	Schneider LS *et al.*	Effectiveness of atypical antipsychotic drugs in patients with Alzheimer's disease.(2006)	*NEJM* (176.076)
7	20	Lyketsos CG *et al.*	Neuropsychiatric symptoms in Alzheimer's disease. (2011)	*Alzheimers Dement* (16.655)
8	19	Geda YE *et al.*	Neuropsychiatric symptoms in Alzheimer's disease: past progress and anticipation of the future. (2013)	*Alzheimers Dement* (16.655)
9	19	Kales HC *et al.*	Management of behavioral and psychological symptoms in people with Alzheimer's disease: an international Delphi consensus. (2019)	*International Psychogeriatrics* (7.191)
10	19	Cerejeira J *et al.*	Behavioral and psychological symptoms of dementia. (2012)	*Frontiers in Neurology* (4.086)

**Table 5:** Top 10 keywords in terms of frequency in the research.

**Ranking**	**Keyword**	**Frequency**
1	Alzheimer’s disease	589
2	Psychological symptom	287
3	Dementia	275
4	Neuropsychiatric symptom	248
5	Prevalence	115
6	Mild cognitive impairment	110
7	Double-blind	107
8	Depression	102
9	Behavioral symptom	82
10	Risk	73

**Table 6:** Clusters of co-occurring keywords.

**Cluster**	**Size**	**Silhouette**	**Label(LLR Algorithm)**	**Most Cited Keywords in a Cluster**
0	90	0.597	Psychological intervention	Symptom; quality of life; people
1	76	0.663	Acetylcholinesterase inhibitor	Double-blind; behavioral symptom; neuropsychiatric inventory
2	61	0.786	Frontal lobe dementia	Cognitive impairment; vascular dementia; diagnostic criteria
3	59	0.624	Down syndrome scale	Prevalence; risk; diagnosis
4	47	0.732	Early marker	Psychological symptom; amyloid beta; mouse model
5	42	0.853	Frontotemporal dementia	Alzheimer’s disease; psychological symptom; dementia
6	38	0.746	Comprehensive management	Risk factor; elderly patient; death
7	37	0.793	Atypical antipsychotics	Agitation; behavioral disturbance; psychiatric symptom
8	28	0.795	Preclinical stage	Psychosis; predictor; cognitive decline
9	24	0.822	Daily living	Impairment; inventory; progression
10	7	0.998	Novel	Aniracetam metabolite; deficit hyperactivity disorder; cerebellar granule cell
11	6	0.997	Gabaergic system	Cognitive deficit; cholinergic hypothesis; choline acetyltransferase

## Data Availability

Not applicable.

## LIST OF ABBREVIATIONS

AD: Alzheimer's Disease
BPSDs: Behavioral and Psychological Symptoms
RCTs: Randomized Controlled Trials
ECT: Electroconvulsive Therapy
TMS: Transcranial Magnetic Stimulation
NET: Nonconvulsive Electrotherapy
rTMS: Repetitive Transcranial Magnetic Stimulation
